# Development and validation of a delirium risk assessment tool in older patients admitted to the Emergency Department Observation Unit

**DOI:** 10.1007/s40520-021-01792-4

**Published:** 2021-02-09

**Authors:** A. Zucchelli, R. Apuzzo, C. Paolillo, V. Prestipino, S. De Bianchi, G. Romanelli, A. Padovani, A. Marengoni, G. Bellelli

**Affiliations:** 1 Department of Information Engineering, University of Brescia, v. Branze, 38, 25123 Brescia, Italy; 2 SC Medicina Generale, Ospedale Sant’Andrea di Vercelli, ASL VC, Vercelli, Italy; 3 Emergency Department, ASST Spedali Civili di Brescia, Brescia, Italy; 4 Department of Clinical and Experimental Medicine, University of Messina, Messina, Italy; 5 Emergency Department, Desio Hospital, ASST Monza e Brianza, Desio, Italy; 6 Department of Clinical and Experimental Science, University of Brescia, Brescia, Italy; 7 University of Milano-Bicocca, Milan, Italy; 8 Geriatric Unit, San Gerardo Hospital, Monza, Italy

**Keywords:** Delirium, Prediction score, Older persons, Emergency department

## Abstract

**Background:**

Delirium is frequent though undetected in older patients admitted to the Emergency Department (ED).

**Aims:**

To develop and validate a delirium risk assessment tool for older persons admitted to the ED Observation Unit (OU).

**Methods:**

We used data from two samples of 65 + year-old patients, one admitted to the ED of Brescia Hospital (*n* = 257) and one to the ED of Desio Hospital (*n* = 107), Italy. Data from Brescia were used as training sample, those collected in Desio as testing one. Delirium was assessed using the 4AT and patients’ characteristic were retrieved from medical charts. Variables found to be associated with delirium in the training sample were tested for the creation of a delirium risk assessment tool. The resulting tool’s performances were assessed in the testing subsample.

**Results:**

Of all possible scores tested, the combination with the highest discriminative ability in the training sample included: age ≥ 75 years, dementia diagnosis, chronic use of neuroleptics, and hearing impairment. The delirium score exhibited an AUC of 0.874 and 0.893 in the training and testing samples, respectively. For a 1-point increase in the score, the odds of delirium increased more than twice in both samples.

**Discussion:**

We propose a delirium risk assessing tool that includes variables that can be easily collected at ED admission and that can be calculated rapidly.

**Conclusion:**

A risk assessment tool could help improving delirium detection in older persons referring to ED.

**Supplementary Information:**

The online version contains supplementary material available at 10.1007/s40520-021-01792-4.

## Background

Delirium is a neuropsychiatric disorder, characterized by acute onset and fluctuating course, change in awareness, arousal, and other cognitive dysfunctions [1]. Delirium is a frequent feature of many acute medical conditions or drug’s intoxication and increases several negative outcomes, including mortality, morbidity, functional impairment [2, 3], and distress of patients and caregivers [4, 5].

The availability of simple scores to predict delirium occurrence in first-intervention settings, such as the Emergency Department (ED), may be particularly useful. Indeed, prompt recognition of delirium is crucial: its misrecognition can lead to inadequate diagnostic evaluations, inappropriate dispositions and diagnosis delays [6, 7] and delirium that is missed in the ED is frequently missed in the acute hospital wards too [8, 9].

However, to date, there are only two studies that have proposed non-externally validated scores to predict delirium in ED [10, 11].

The aim of this study was to develop and validate a delirium score for older persons admitted to the ED Observation Unit (OU).

## Methods

We retrospectively collected data from two samples of patients, one admitted to the ED of the Civili Hospital in Brescia (*n* = 257) and the other to the ED of the Desio Hospital (Monza) (*n* = 107), Italy. Data were collected between November 2018 and February 2019 in Brescia and between July and September 2019 in Desio. To decrease the risk of overfitting and to test the generalizability of the proposed score, the first sample (Brescia) was employed to build the delirium risk assessment tool (training sample), whereas the second one (Desio) was used to externally validate the chosen score and to test its accuracy (testing sample).

The only inclusion criterium was age equal or above 65 years; exclusion criteria were coma, end-stage dementia and inability to speak Italian.

Among patients evaluated, 9 were excluded for missing information on most of the variables.

The study was approved by the Ethical Committee of the Brescia County and the one of Monza.

### Diagnosis of delirium

Presence of delirium was assessed by the attending physician and a resident in geriatrics using the 4AT test [12]. The 4AT has shown excellent sensitivity and specificity to diagnose delirium at a threshold score of ≥ 4 [12, 13]. The 4AT was repeated every eight hours: delirium was considered present if a score ≥ 4 was exhibited in at least one evaluation.

### Data collection

The following data were retrieved from medical charts: reason for ED admission, vital parameters, presence of visual and/or hearing loss, and chronic conditions. A patient was considered affected by dementia if an established diagnosis of dementia was reported in his/her medical history. The pain was measured with a numerical rating scale. Laboratory tests were performed in each patient.

Presence of “suspected infection” was based on the reason of ED admission and/or C-reactive protein levels higher than 5 mg/L.

Patients’ chronic therapy was retrieved from medical records and in categories. Antiepileptic drugs, antipsychotics, antidepressants (SNRI, SSRI, atypical, tricyclics), anti-dementia drugs, hypnotics, opioids, and drugs for Parkinson disease were grouped in a single category named “psychotropics”.

The total length of stay in OU was recorded as well as the number of patients who spent 2 + hours in OU between 06:00 pm and 06:00 am.

### Statistical analyses

The differences between the training (Brescia’s ED) and the testing (Desio’s ED) sample were investigated employing chi-squared tests or exact e Fisher’s tests and *t*-tests or Mann–Whitney tests, as appropriate. The association between patient’s characteristics and delirium was explored by means of unadjusted logistic regression models in the training sample. We used a *p* ≤ 0.200 cut-off to select the variables associated with delirium to be tested for the creation of the delirium risk assessment tool. Continuous variables associated with delirium were dichotomized using a clinically suitable cut-off.

The following principles were used to create the score: (1) the final tool should have included between 2 and 4 variables and (2) a score between 1 and 3 could have been assigned to each included variable. All possible combinations of variables, number of variables included, and points assigned were tested. The discriminative ability to predict delirium in the training sample was evaluated using the area under the curve (AUC) obtained from non-parametric Receiver-Operating-Characteristic (ROC) analyses. The combination showing the highest AUC in the training sample was selected and the performances of the resulting score were assessed in the testing sample. All analyses were conducted with R 4.0.0 (R Core Team—R Foundation for Statistical Computing, Vienna, Austria).

## Results

The characteristics of the training and testing samples are described in Table S1. The proportion of patients who were diagnosed with delirium was 16.1% in the training sample and 34.6% in the testing sample. In the latter, patients were older, more likely to have dementia, more likely to be admitted for a suspected infectious disease and spent more time in the OU in comparison with those in the training sample.

**Fig. 1:**
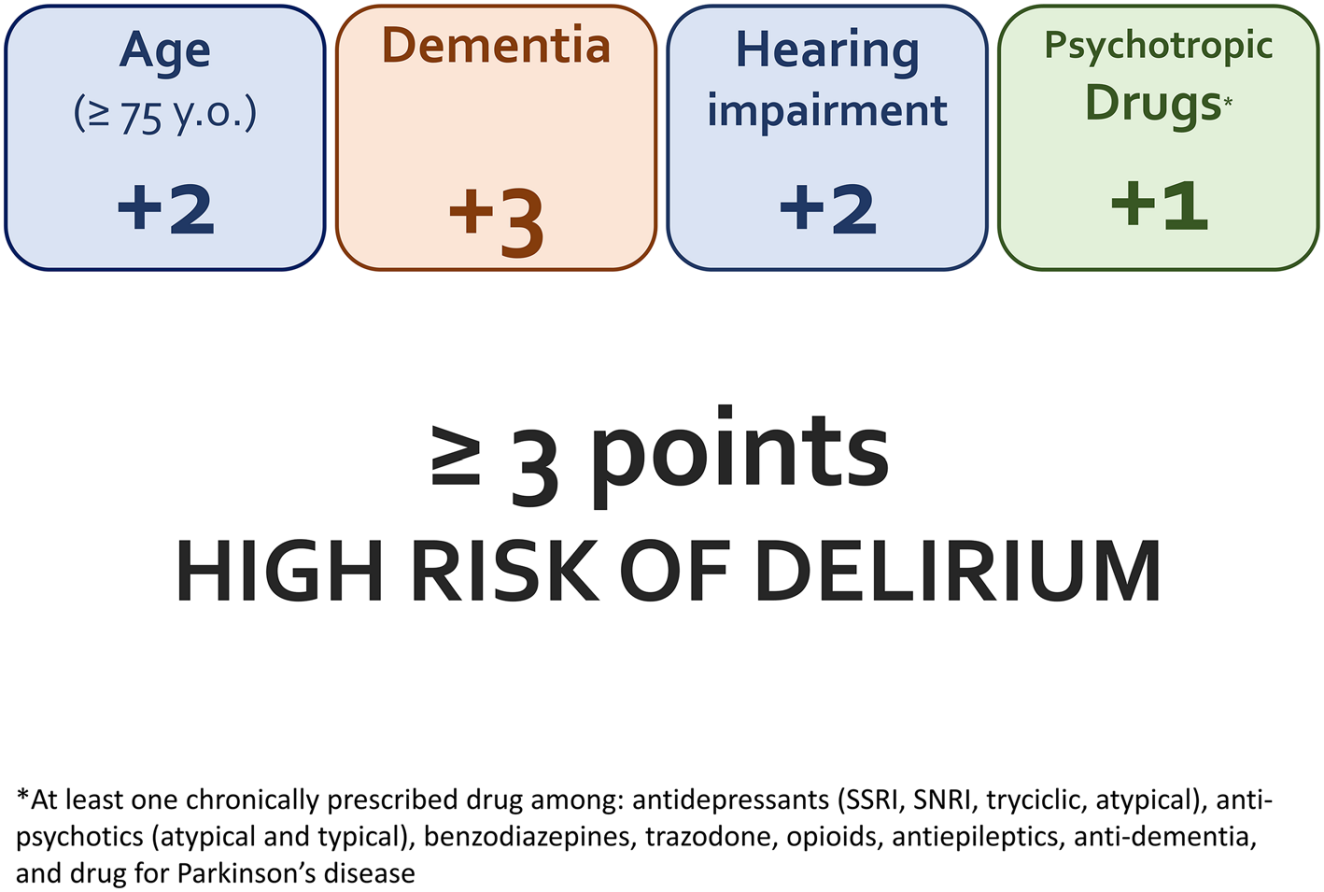
Summary of the components of the delirium risk assessment tool

Seven variables were found to be associated with delirium in the training sample and were considered for the creation of the delirium score (Table 1). When evaluated alone, each drug class included in the “psychotropics” group was found positively associated with delirium, although 4 of them (i.e. antiepileptics − proportion = 2.5%, atypical antidepressants  − 0.8%, opioids  − 6.1%, and tricyclics − 1.1%) did not reach statistical significance (data not shown). In total, 3969 possible scores were tested in the training sample. The combination that lead to the highest discriminative ability in the training sample included age ≥ 75 years old (2 points), dementia diagnosis (3 points), chronic use of psychotropic drugs (1 point), and hearing impairment (2 point) (Fig. 1). In both samples, the score ranged between 0 and 8 points and the median value was 2 points.

**Table 1:** Associations (odds ratios—OR—and 95% confidence intervals—95%CI) of patients’ characteristics with delirium development and their area under the curve (AUC), in the training sample

	OR (95%CI)	*p*	AUC
Age	1.12 (1.06–1.17)	< 0.001	0.733
Age ≥ 75 years old	9.22 (3.20–39.06)	< 0.001	0.676
Male sex	0.89 (0.44–1.76)	0.744	0.514
Living in nursing home	2.03 (0.43–7.38)	0.313	0.518
Dementia	18.33 (8.08–43.64)	< 0.001	0.744
Hearing impairments	8.07 (3.80–17.44)	< 0.001	0.699
Visual impairments	1.03 (0.45–2.21)	0.932	0.503
Anticoagulant drugs	1.4 (0.61–3.02)	0.406	0.529
PPIs	1.47 (0.74–2.98)	0.272	0.548
Insulin	1.42 (0.45–3.81)	0.511	0.517
NSAIDs	1.32 (0.19–5.5)	0.735	0.506
Opioids	1.61 (0.35–5.54)	0.488	0.513
Psychotropics	5.55 (2.68–12.23)	< 0.001	0.728
Suspected Infection	2.23 (1.12–4.45)	0.022	0.597
Cardiovascular event	1.56 (0.73–3.21)	0.234	0.545
Stroke	5.31 (0.21–136.13)	0.241	0.51
Acute urinary retention	0.38 (0.02–2.02)	0.364	0.519
Heart rate	1.00 (0.98–1.02)	0.899	0.503
Systolic blood pressure	0.98 (0.96–0.99)	0.010	0.628
Systolic blood pressure < 120 mmHg	2.49 (0.98–6.00)	0.049	0.554
Peripheral arterial blood oxygen saturation	1.00 (0.9–1.16)	0.976	0.531
Pain	1.00 (0.89–1.13)	0.938	0.506
Haemoglobin < 10 g/dL	1.25 (0.34–3.61)	0.704	0.509
Creatinine	1.1 (0.81–1.43)	0.467	0.624
Sodium	0.94 (0.87–1.01)	0.092	0.630
Sodium < 135 mEq/L	2.54 (0.92–6.47)	0.057	0.549
Total time spent	1.01 (0.97–1.04)	0.660	0.529
2 + hours spent between 06:00 pm and 06:00 am	1.84 (0.68–6.44)	0.274	0.535

Variables with a* p* < = 0.200 were tested for the creation of the delirium risk assessment score

*PPIs *proton pumps inhibitors, *NSAIDs *non-steroidal anti-inflammatory drugs

**Fig. 2:**
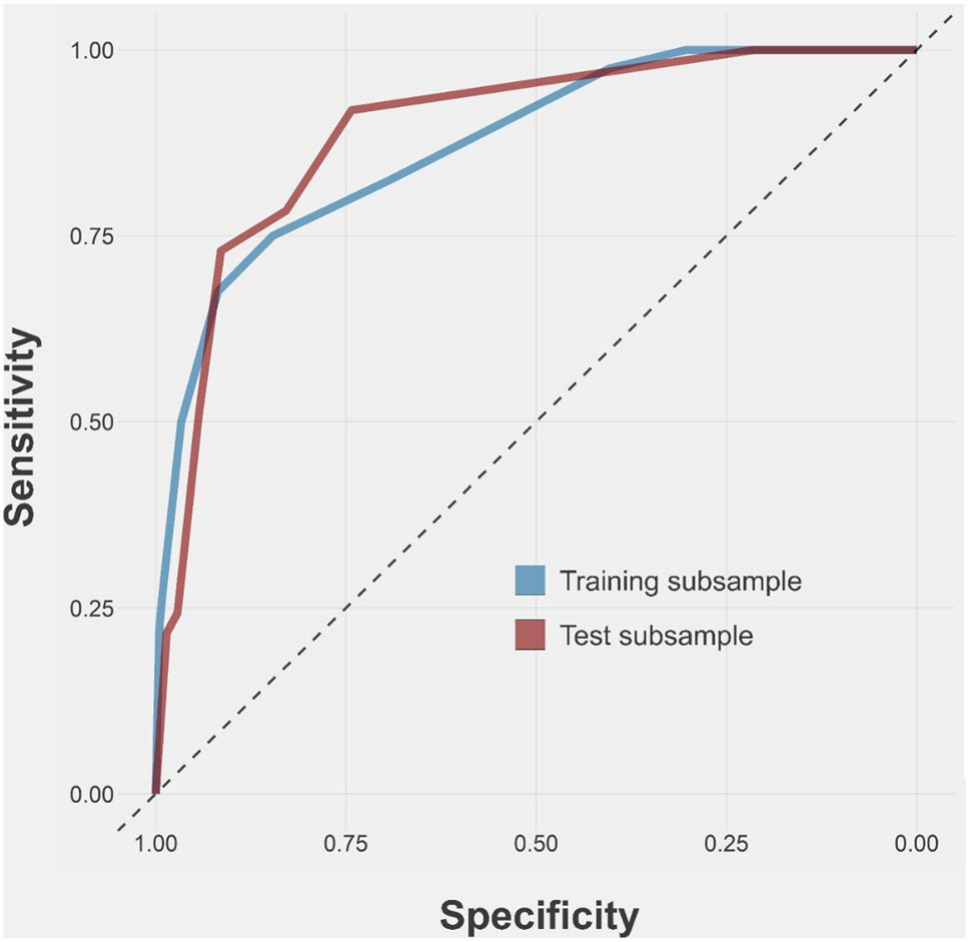
Delirium predictive score’s ROC curves (training and testing samples)

Figure 2 and Table S2 show the performance of the delirium score. The score exhibited an AUC of 0.874 and 0.893 in the training and testing samples, respectively. In both samples, a cut-off of 3 or more points identified delirium with a sensitivity higher than 0.80, while a cut-off of 5 or more points leads to a specificity of 0.90 or higher. For a 1-point increase in the score, the odds of developing delirium increased more than twice in both samples [training sample OR (95%CI): 2.22 (1.80–2.84); testing sample OR (95%CI): 2.35 (1.78–3.30)]. After excluding patients with a diagnosis of dementia, the odds ratios of developing delirium for a 1-point increase were 2.17 (95%CI: 1.53–3.22) and 2.70 (95%CI: 1.52–5.37) in the training and testing sample, respectively.

## Discussion

We developed and validated a delirium risk assessment tool using retrospective data from two different samples of older patients admitted to the ED OU. Variables included in the score were age ≥ 75 years old, dementia, hearing impairment, and chronic use of psychotropic drugs. In both samples, a cut-off of 3 or more points identified delirium with a sensitivity higher than 0.80, while a cut-off of 5 or more points lead to a specificity of 0.90 or higher.

Age, dementia, and hearing impairment have been shown to be associated with delirium in previous studies [14, 15]. Further, several drugs of those included in the “psychotropics” group have been directly associated with an increased risk of delirium in the literature [16, 17]. However, it is also likely that the chronic prescription of psychotropic drugs reflects the presence of conditions that may increase the chances of delirium, such as previous episodes of delirium, cognitive impairment or dementia, explaining the increase in the discriminative capacity of the score after the inclusion of chronic psychotropic therapy among the variables.

Other scores have been proposed. The delirium risk score by Han et al. [18] was validated against the Confusion Assessment Method (CAM) for the Intensive Care Unit [19] demonstrating good performance (AUC = 0.82) and included three variables: hearing impairment, dementia, and Katz-Activity of Daily Living (ADL). The risk prediction rule by Kennedy et al. [10] consisted of 5 variables (i.e. older age, history of stroke, dementia, suspected infection, tachypnea, and intracranial hemorrhage) and demonstrated fair prediction ability towards a CAM-defined diagnosis of delirium (AUC = 0.77). The score by Pendlebury et al. [20] was created to identify both prevalent and incident delirium in acute wards and included older age, cognitive impairment, severe illness, infection, and visual impairment. The AUC of this score was 0.78 for any form of delirium, as defined with the CAM and DSM-IV criteria. Limits of these scores are that they were not externally validated [10], that delirium was assessed only at a single time-point in the ED [10] and that they require additional scales to be computed [11]. Furthermore, two studies used delirium diagnostic tools that are not applicable in drowsy patients [10, 20] which may have resulted in an underestimation of hypoactive delirium, which is the commonest subtype in ED [11].

The delirium score we propose has several advantages; first, it includes variables that can be easily collected at ED admission by the attending physician or nurse irrespectively of his/her expertise. Second, the score can be calculated rapidly, characteristic that is of most importance in critical care settings.

Given the frequent underdiagnosis of delirium in the EDs [11], a delirium risk assessment tool could help to improve delirium detection in older persons referring to acute care. The early identification of persons at increased risk of developing delirium in OU may help to put in place preventive strategies to decrease the chances of delirium, as well as to prompt a comprehensive geriatric assessment that may help to recognize possible triggers and causes of delirium.

### Limitations

The results of our study should be read in light of some limitations: first, data about the timing of delirium development (i.e. as presentation of an acute disease, during the evaluation in the ED or during the OU staying) were not available; however, all variables included in our score were retrieved from the past medical history of the patient and are not subject to delirium onset’s timing; furthermore, we selected the combination of variables showing the highest AUC in the training dataset, increasing the risk of overfitting: however, the association of all selected variables with delirium is known and the score was validated in another sample. Future studies are needed in order to investigate the causal relationship between the variables included in our tool and delirium; lastly, dementia alone showed a high discriminative ability in the prediction of delirium, however almost 40% of the patients developing delirium missed a previous dementia diagnosis and our score was strongly associated with delirium development in patients without known cognitive impairment.

## Conclusion

A simple and rapid risk assessment tool could help improving delirium detection in older persons referring to ED.

## Supplementary Information

Below is the link to the electronic supplementary material.

Supplementary file1 (DOCX 17 KB)

Supplementary file2 (DOCX 15 KB)

## Data Availability

Access to the original data is available to the research community upon approval by the data management committee. Application for accessing data can be sent to: alessandra.marengoni@unibs.it and giuseppe.bellelli@unimib.it.
