# Supplementary material for: Development and validation of a delirium risk assessment tool in older patients admitted to the Emergency Department Observation Unit
Source: Aging Clin Exp Res. 2021 Feb 9;33(10):2753–8. doi: 10.1007/s40520-021-01792-4 (PMC8531045; doi:10.1007/s40520-021-01792-4)
Supplement: Supplementary file 1 — Supplementary file1 (DOCX 17 KB) [file 40520_2021_1792_MOESM1_ESM.docx]

**Table S1**. Characteristics of the training and test samples

|  |  | Training sample  N = 248 | Testing sample  N = 107 | *p* |
| --- | --- | --- | --- | --- |
| *Demographics* | | | | |
| Age, y - mean (SD) |  | 77.8 (7.7) | 83.3 (7.4) | <0.001 |
| Male sex – N (%) |  | 105 (42.3) | 46 (43.0) | 1.000 |
| Living in nursing home – N (%) |  | 11 (4.4) | 1 (0.9) | 0.117 |
| *Chronic conditions* | | | | |
| Dementia – N (%) |  | 35 (14.1) | 30 (28.0) | 0.003 |
| Hearing impairments – N (%) |  | 44 (17.8) | 22 (20.6) | 0.645 |
| Visual impairments – N (%) |  | 62 (25.1) | 34 (31.8) | 0.243 |
| *Chronic therapy* | | | | |
| Anticoagulant drugs – N (%) |  | 50 (20.2) | 30 (28.3) | 0.124 |
| PPIs – N (%) |  | 129 (52.0) | 53 (50.0) | 0.817 |
| Insulin – N (%) |  | 24 (9.7) | 13 (12.1) | 0.590 |
| NSAIDs – N (%) |  | 10 (4.0) | 1 (0.9) | 0.230 |
| Opioids – N (%) |  | 13 (5.2) | 10 (9.4) | 0.219 |
| Psychotropics – N (%) |  | 96 (38.7) | 37 (34.6) | 0.536 |
| *Reasons for ED admittance* | | | | |
| Suspected infection – N (%) |  | 32 (12.9) | 44 (41.1) | <0.001 |
| Cardiovascular event – N (%) |  | 60 (21.6) | 3 (3.9) | <0.001 |
| Stroke – N (%) |  | 2 (0.8) | 1 (0.9) | 1.000 |
| Acute urinary retention – N (%) |  | 14 (5.6) | 0 (0.0) | <0.001 |
| *Vital signs* | | | | |
| Heart rate, bpm – mean (SD) |  | 76.42(12.2) | 68.6 (13.4) | <0.001 |
| Systolic Blood Pressure, mmHg – mean (SD) |  | 145.8 (22.4) | 133.9 (22.6) | <0.001 |
| Peripheral arterial blood oxygen saturation, % - median (IQR) |  | 97.0 (3.0) | 95.0 (4.0) | <0.001 |
| Pain, NRS – median (IQR) |  | 0.0 (4.0) | 0.0 (0.0) | 0.029 |
| *Blood tests* | | | | |
| Haemoglobin, g/dL – mean (SD) |  | 12.5 (1.9) | 11.6 (2.0) | <0.001 |
| Creatinine, mg/dL – median (IQR) |  | 1.0 (0.6) | 1.4 (0.9) | <0.001 |
| Sodium, mEq/L – mean (SD) |  | 139.9 (4.6) | 139.9 (6.8) | 0.973 |
| *Observation area stay* | | | | |
| Delirium – N (%) |  | 40 (16.1) | 37 (34.6) | <0.001 |
| Total time spent, hours – median (IQR) |  | 17.0 (10.0) | 20.4 (14.1) | <0.001 |
| 2+ hours spent between 06:00 pm and 06:00 am – N (%) |  | 207 (84.1) | 98 (92.5) | 0.053 |

Abbreviations: SD = standard deviation; PPIs = Proton Pumps Inhibitors; NSAIDs = Non-steroidal Antinflammatory Drugs; bpm = beats per minute
Missing: 6 for haemoglobin, 5 for creatinine, 2 for sodium
