# Supplementary material for: Development and validation of a delirium risk assessment tool in older patients admitted to the Emergency Department Observation Unit
Source: Aging Clin Exp Res. 2021 Feb 9;33(10):2753–8. doi: 10.1007/s40520-021-01792-4 (PMC8531045; doi:10.1007/s40520-021-01792-4)
Supplement: Supplementary file 2 — Supplementary file2 (DOCX 15 KB) [file 40520_2021_1792_MOESM2_ESM.docx]

**Table S2**. Specificity, sensitivity, positive predictive value (PPV), and negative predictive value (NPV) of the possible cut-offs of the delirium predictive score in the training and testing samples.

|  | Training sample  (AUC = 0.874) | | | | TestING sample  (AUC = 0.893) | | | |
| --- | --- | --- | --- | --- | --- | --- | --- | --- |
| Cut-off | specificity | sensitivity | PPV | NPV | specificity | sensitivity | PPV | NPV |
| ≥ 1 | 0.303 | 1.00 | 0.216 | 1.000 | 0.186 | 1.000 | 0.394 | 1.000 |
| ≥ 2 | 0.404 | 0.975 | 0.239 | 0.988 | 0.214 | 1.000 | 0.4024 | 1.000 |
| ≥ 3 | 0.692 | 0.825 | 0.340 | 0.954 | 0.743 | 0.919 | 0.654 | 0.945 |
| ≥ 4 | 0.846 | 0.750 | 0.484 | 0.946 | 0.829 | 0.784 | 0.7074 | 0.879 |
| ≥ 5 | 0.918 | 0.675 | 0.614 | 0.936 | 0.914 | 0.730 | 0.8184 | 0.865 |
| ≥ 6 | 0.966 | 0.500 | 0.741 | 0.910 | 0.943 | 0.514 | 0.8264 | 0.786 |
| ≥ 7 | 0.990 | 0.275 | 0.846 | 0.877 | 0.971 | 0.2434 | 0.8184 | 0.708 |
| 8 | 0.995 | 0.225 | 0.900 | 0.870 | 0.986 | 0.2164 | 0.889 | 0.704 |
